# Demonstration of an algorithm to overcome health system-related barriers to timely diagnosis of breast diseases in rural Zambia

**DOI:** 10.1371/journal.pone.0196985

**Published:** 2018-05-10

**Authors:** Leeya F. Pinder, Jean-Baptiste Nzayisenga, Aaron Shibemba, Victor Kusweje, Hector Chiboola, Mary Amuyunzu-Nyamongo, Sharon Kapambwe, Catherine Mwaba, Pavlo Lermontov, Chibamba Mumba, Ronda Henry-Tillman, Groesbeck P. Parham

**Affiliations:** 1 Department of Obstetrics and Gynecology, Division of Global Women’s Health, University of North Carolina at Chapel Hill, Chapel Hill, NC, United States of America; 2 Department of Obstetrics and Gynecology, Women and Newborn Hospital, Lusaka Zambia, Lusaka, Zambia; 3 Department of Pathology, Kabwe General Hospital, Kabwe, Zambia; 4 Department of Social Sciences, Chreso University, Lusaka, Zambia; 5 African Institute for Health and Development, Nairobi, Kenya; 6 Ministry of Health, Lusaka, Zambia; 7 Cancer Diseases Hospital, Lusaka, Zambia; 8 Department of Surgery, University of Arkansas for Medical Sciences, Little Rock, AR, United States of America; University of Nebraska-Lincoln, UNITED STATES

## Abstract

**Background:**

Long delays to diagnosis is a major cause of late presentation of breast diseases in sub-Saharan Africa.

**Aims:**

We designed and implemented a single-visit breast care algorithm that overcomes health system-related barriers to timely diagnosis of breast diseases.

**Methods:**

A multidisciplinary team of Zambian healthcare experts trained a team of mid- and high-level Zambian healthcare practitioners how to evaluate women for breast diseases, and train trainers to do likewise. Working collaboratively, the two teams then designed a clinical platform that provides multiple breast care services within a single visit. The service platform was implemented using a breast outreach camp format, during which breast self-awareness, psychosocial counseling, clinical breast examination, breast ultrasound, ultrasound-guided biopsy, imprint cytology of biopsy specimens and surgical treatment or referral, were offered within a single visit.

**Results:**

Eleven hundred and twenty-nine (1129) women attended the camps for breast care. Mean age was 35.9 years. The majority were multiparous (79.4%), breast-fed (76.0%), and reported hormone use (50.4%). Abnormalities were detected on clinical breast examination in 122 (10.8%) women, 114 of whom required ultrasound. Of the 114 who underwent ultrasound, 48 had identifiable lesions and were evaluated with ultrasound-guided core needle biopsy (39) or fine-needle aspiration (9). The concordance between imprint cytology and histopathology was 100%, when breast specimens were classified as either benign or malignant. However, when specimens were classified by histopathologic subtype, the concordance between imprint cytology and histology was 85.7% for benign and 100% for malignant lesions. Six (6) women were diagnosed with invasive cancer. Eighteen (18) women with symptomatic breast lesions had next-day surgery.

**Significance:**

Similar to its impact on cervical cancer prevention services, a single visit breast care algorithm has the potential to overcome health system-related barriers to timely diagnosis of breast diseases, including cancer, in rural African settings.

## Introduction

Globally, the incidence of breast cancer is projected to surge over the next two decades, largely due to increases in ageing populations, reductions in mortality from infectious diseases and shifts in lifestyle risk factors, such as reproductive patterns, tobacco use and obesity [1–4]. Approximately 70% of new cases will occur in low- and middle-income countries (LMICs) [4] where health systems are commonly characterized by underfunding and scarce mid- and high-level human resources. Women living in sub-Saharan Africa (SSA) will be profoundly affected, as most typically present with late stage disease, resulting in 5-year relative survival rates of < 50% (2–4). Those residing in rural Africa have an even higher percentage of late stage disease than their urban counterparts [5–7]. Zambia, a country already heavily burdened with HIV and cervical cancer [8–10], is predicted to have a 25% increase in incident breast cancer cases and deaths by the year 2020 [9].

Having made cancer control a national priority, the Zambian Ministry of Health avidly supports and facilitates the implementation of contextually appropriate and sustainable approaches to early detection and treatment [11]. In 2005, the burden of cervical cancer among previously unscreened women in Zambia was addressed through the establishment of a public-sector “screen and treat” prevention program [12] that links screening, using visual inspection with acetic acid (VIA), to immediate treatment of precancerous lesions with either cryosurgery, thermocoagulation or electrosurgical excision. Intervening histologic evaluation is reserved for cases of suspected cancer. This WHO-approved single-visit approach to cervical cancer prevention in resource-constrained environments avoids delays in diagnosis and treatment, and significantly reduces loss to follow-up, in contrast to the prolonged, multi-step Pap smear/HPV-colposcopy-biopsy-histologic assessment-treatment algorithm commonly used in high-income settings.

To date, over 500,000 women have been screened throughout Zambia using this method. Central to its success are the immediacy of test results and compression of the prevention pathway into a single visit. Using lessons learned from the cervical cancer prevention experience, we sought to improve time to diagnosis through the design and implementation of a single-visit algorithm for breast care.

## Methods

The breast care clinical pathway is a multi-step process that involves awareness–screening–radiographic imaging–cytohistologic examination–treatment. Long delays before, at or between each step can significantly contribute to advanced presentations of both benign and malignant diseases [13]. We designed an algorithm that compresses the multi-step pathway into a single visit, during which the following services are made available: (1) breast self-awareness, (2) lay psychosocial counseling, (3) clinical breast examination (CBE), (4) breast ultrasound, (5) ultrasound-guided breast biopsy, as indicated, (6) imprint cytology of breast biopsy specimens, (7) post-cytology counseling, and (8) immediate surgical treatment or referral. The algorithm was implemented within the context of two separate, week-long, breast care outreach camps inside government-operated health facilities located in a predominantly rural (Central) province.

### Preparatory activities

The preparatory phase of the project involved assembling local healthcare experts from multiple disciplines–health promotion, lay counseling, nursing, radiology, pathology, general surgery–as well as stakeholders from civil society (religious leaders, women’s health advocates, traditional healers, etc.), over a two-week period, to develop a breast care manual. The manual was designed to equip the user with relevant competencies in breast care, inclusive of breast cancer control. Once completed, the manual was used by the cadre of local experts (Master Trainers) to train others (Training of Trainers), who in turn trained local healthcare providers who were located at the proposed rural outreach sites. The trainees that were trained by the Master Trainers consisted of four peer educators (health promotion), two lay counselors (lay psychosocial counseling), four nurses (clinical breast examination), two radiographers (breast ultrasound), two general medical officers (ultrasound-guided biopsy of the breast) and two cytohistology technicians (imprint cytology). Two local general surgeons had previously been trained in modern methods of surgical management of breast abnormalities under another Susan G. Komen-sponsored program [14].

One week prior to the outreach camps, peer educators travelled throughout the catchment areas (Kapiri Mposhi District–pop. 303,263; Kabwe District—pop. 202,914) in the Central Province, raising awareness through messaging that (a) stressed the importance of breast health, (b) listed the common signs and symptoms of breast cancer, and (c) provided the dates and location of the event. Information leaflets were handed out and public announcements were made using a mobile PA system targeting strategic sites such as local markets and residential areas. Radio interviews with peer educators were conducted, coupled with continuous radio announcements detailing the upcoming event. All messaging was conducted in English and local languages.

### Single visit breast care outreach camp

**Fig 1:**
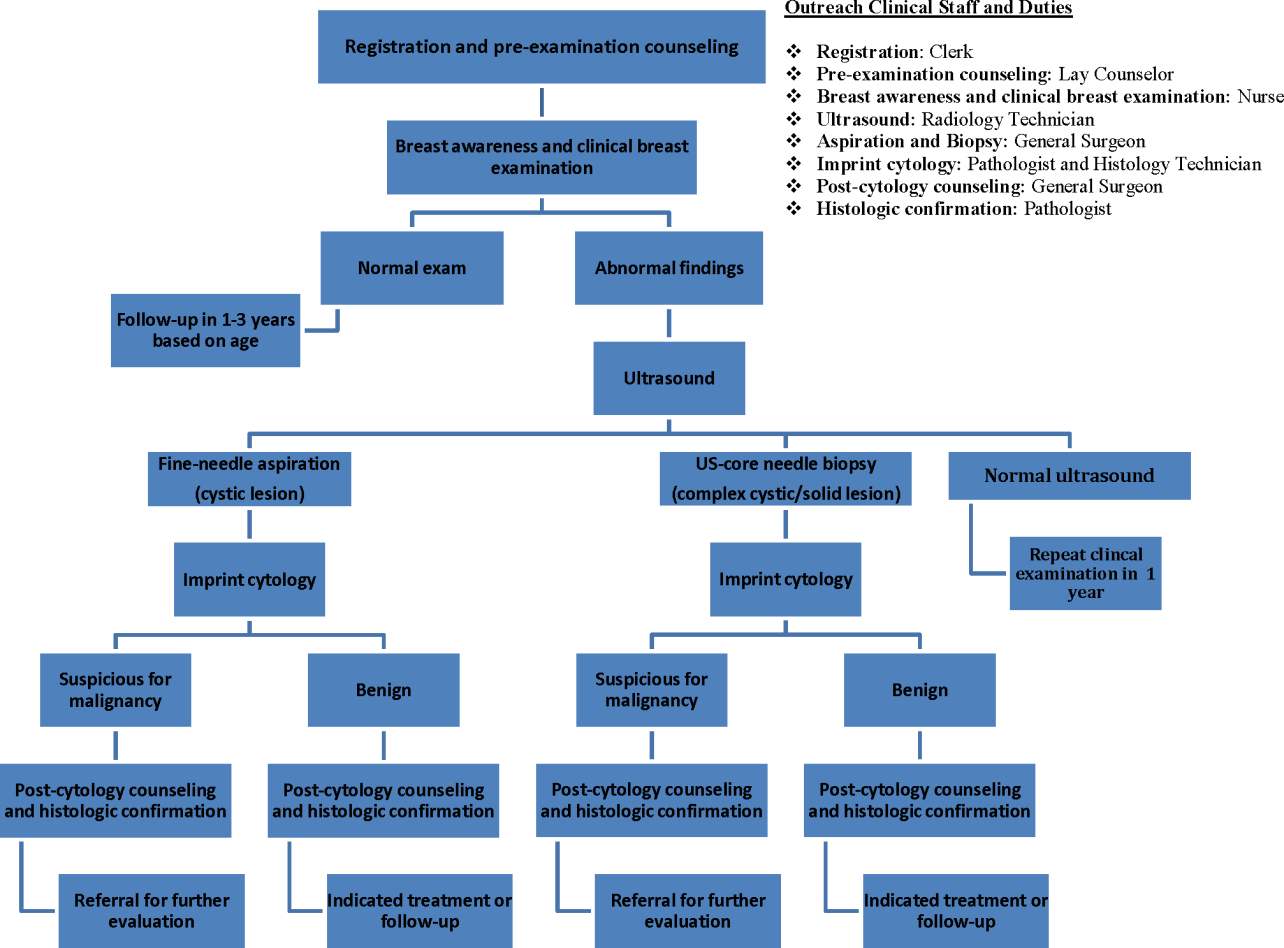
Breast care model flow diagram.

The breast care camps were conducted at government-operated heath facilities, one a primary health clinic (Kapiri Mposhi Urban Clinic), and the other a provincial district hospital (Kabwe General Hospital). During the camps, all service components in the breast care pathway were bundled into a single visit format, including an option for surgery, if indicated. (Fig 1). Each service was offered in separate, but adjoining rooms, in the following manner: Step 1—Pre-Counseling–conducted by lay counselors to explain to women the intent and value of the various services that were being offered; to emphasize the importance of knowing what is normal for their breasts as a component of breast self-awareness; and to address any questions, fears, myths and misunderstandings women might have. Step 2—Clinical Breast Examination–conducted by nurses who provided instructions on how to perform self-breast examination, as part of breast self-awareness, and information on the common signs and symptoms of breast cancer. Women found to have breast abnormalities were immediately navigated to the Breast Ultrasound Room for evaluation. Step 3—Breast Ultrasound and Ultrasound-guided Core Biopsy/Needle Aspiration–performed by radiographers and general medical officers/general surgeons, respectively, following written informed consent by the woman. Step 4—Imprint Cytology–conducted by cytohistology technicians who obtained cytology specimens from the fresh breast core biopsies or aspirates and made cytology slides (imprint cytology) onsite, which were immediately interpreted by the camp pathologist. Cytology results were given to the participant by the general medical officer/general surgeon, with the understanding that it was a provisional diagnosis. Each afternoon, specimens previously fixed in formalin were delivered to the nearest pathology lab (Kabwe General Hospital) where they were rapidly processed for final histologic diagnosis. One day later, histologic analyses were made available and final results given to women by the physician who performed the biopsy. Step 5—Lay Counseling–provided again, but this time to women diagnosed with breast abnormalities that either required some form of treatment, or observation. The primary purpose was to stress the importance of follow-up at Kabwe General Hospital and to assist in the organization of transportation. Step 6—Breast Surgery–offered to those in whom abnormal histopathology was confirmed, surgical resection was indicated, and could be safely and effectively accomplished at the local medical facility. Written consent was obtained prior to the surgical procedure being performed.

## Results

Eleven hundred and twenty-nine (1129) women attended the two camps and all received breast health counseling and clinical breast examination. Mean age of camp participants was 35.9 years (±13.0). HIV positivity was 19% (228). The majority (79.4%) had at least one pregnancy and breastfed (76.0%), while half (50.4%) reported hormone use, the majority of which were estrogen-containing products (71.6%) (Table 1). Breast abnormalities were clinically detected in 122 (10.8%) women (Table 2) of which 114 were referred for evaluation with ultrasound. Of the 114 evaluated with ultrasound, 53 (46.5%) had normal findings and were counselled to repeat CBE in 1 year or earlier if symptomatic. Fifty-nine (51.8%) women underwent either ultrasound-guided core needle biopsy for solid-appearing lesions (39), fine-needle aspiration for cystic-appearing lesions (9), local excision for lesions not amenable to needle biopsy (7), or cyst drainage (5) (Table 2). One woman had two separate lesions that required biopsy and cyst drainage. Another, age 15 years, was not desirous of further diagnostic evaluation of a palpable breast mass. Data are missing on one participant. All specimens obtained through biopsy or aspiration were immediately processed, on-site, for imprint cytology and confirmed histologically in the referral pathology lab. Concordance between cytology and histology was 100% when classifying breast lesions as benign or malignant. Concordance between cytology and histology was 85.7% for benign lesions and 100% for malignant lesions, when classifying them according to histopathologic subtype. Fibroadenoma (15) was the most common benign diagnosis (Table 3). Twenty-two (22) women underwent surgical excision for treatment or diagnosis of symptomatic breast lesions. Less than 1% (6/1129) were diagnosed with invasive cancer, all of whom had follow-up care immediately arranged at the nearby provincial hospital (Kabwe General Hospital).

**Table 1:** Demographics of breast camp clients.

*Characteristics*	Kapiri *N = 475*	Kabwe *N = 654*	Total *N = 1129*
**Age (years)**			
Mean age:	34.5 (±13.0)	37.3 (±13.9)	35.9 (±13.5)
Age <35	256 (53.9)	301 (46.0)	557 (49.3)
Age 35–45	125 (26.3)	164 (25.1)	289 (25.6)
Age 45+	94 (19.8)	188 (28.7)	282 (25.0)
**Hormonal status**			
Menopausal	34 (7.2)	60 (9.2)	94 (8.4)
Reported hormone use*	261 (54.9)	309 (46.9)	570 (50.4)
Estrogen-containing	192 (73.6)	214 (69.7)	406 (71.6)
Progesterone only	36 (13.8)	94 (30.6)	130 (22.9)
**Family History**			
Reported family history of breast cancer	17 (3.6)	51 (7.8)	68 (6.0)
**Pregnancy History**			
Women with at least one pregnancy	389 (81.9)	507 (77.5)	896 (79.3)
Mean number of pregnancies	3.4 (±2.8)	4.1 (±2.7)	4 (±2.7)
Mean age at 1^st^ pregnancy	19.6 (±3.6)	19.8 (±4.0)	19.7 (±3.8)
Mean age at 1^st^ delivery	19.9 (±3.5)	20.1 (±4.0)	20.0 (±3.8)
Number who breastfed	373 (95.9)	485 (95.7)	858 (95.8)
**HIV Status**			
HIV (+)	102 (21.5)	116 (17.7)	218 (19.3)
HIV (+) on ART	89 (87.3)	113 (97.4)	202 (92.7)
HIV (+), not on ART	3 (2.9)	3 (2.7)	7 (3.2)
HIV (+), no ART info	10 (9.8)	0 (0)	9 (4.1)
HIV (-)	268 (56.4)	436 (66.6)	707 (62.6)
HIV unknown	105 (22.1)	106 (16.2)	204 (18.1)

*Missing hormone type (Kapiri-33, Kabwe-1)

**Table 2:** Diagnostic outcomes.

*Outcomes*	Kapiri *N = 475*	Kabwe *N = 654*	Total *N = 1129*
**Abnormalities detected on CBE**	*33	^§^89	122
Referred for ultrasound evaluation	32	82	114
**Ultrasound Findings**	**32**	^†^**87**	**119**
Cystic lesion	8	8	16
Solid lesion	9	^†^39	48
Solid/cystic	1	1	2
No lesion seen	14	39	53
**Procedures**	**20**	**51**	**71**
Surgical excision of breast mass	3	19	22
Fine-needle aspirations	5	4	9
US-guided core needle biopsy	12	27	39
Surgical excision of axillary mass	0	1	1

* One woman—repeat clinical breast exam in 3 months

§ Five women—referred to gynecologist for further management; one to return for CBE in 3 months; one received topical treatment for breast skin disorder

† Four women with multiple breast lesions—triaged to observation

**Table 3:** Histologic outcomes.

Histology		
Outcomes:		48
	Cancer	6
	Fibroadenoma	15
	Chronic abscess	4
	Mastitis	4
	Pseudoangiomatous stromal hyperplasia (PASH)	4
	Tubular adenoma	2
	Lipoma	2
	Simple cyst	1
	Galactocele	1
	Usual ductal hyperplasia	1
	Lactational changes	1
	Indeterminate	5
	Missing data	1

### Onsite training

During the course of the single-visit breast care camps, trainers from Lusaka provided the following clinical educational services for local healthcare providers at the rural outreach sites: two cervical cancer prevention nurses were taught how to perform CBE, the breast ultrasound skills and techniques of four radiographers were updated, three general medical officers were mentored in ultrasound-guided core needle biopsy, two cytohistology technicians were trained to perform imprint cytology, and two local lay persons were instructed in lay counseling.

## Discussion

In general, delays from symptom onset to time of diagnosis can potentiate advanced presentations of breast diseases. Where breast cancer is concerned, the evidence is substantial that delays of more than 3 months from symptom recognition to diagnosis is associated with late stage presentation and poorer survival [15]. In sub-Saharan Africa, where breast cancer is commonly characterized by advanced stage (45–90% stage III and IV) at the time of diagnosis [5, 7, 16, 17], and low (≤50%) 5-year survival rates [13, 17, 18], these delays average 6 months or greater [19]. Delays can be further stratified into woman-level (e.g. low educational level, poor socioeconomic status, living in a rural area, poor breast cancer awareness, belief in traditional or spiritual medicine) and health system-related (e.g. distance to nearest healthcare provider, number of healthcare providers visited prior to diagnosis, health professionals with poor breast cancer knowledge, unavailability of suitable diagnostic facilities, lack of appropriate referral pathways) [5, 7, 20].

We implemented a breast care algorithm with the aim of overcoming health system-related barriers to timely diagnosis of breast diseases. Breast health education, clinical breast examination, radiographic imaging, cytohistologic diagnosis and immediate treatment or referral services were offered in a single-visit format. Over 1,000 women attended the two, week-long, breast care outreach camps. While the vast majority (88.9%) did not have a clinically detectable lesion, they all received breast health promotion messages and were taught the importance of breast self-awareness and CBE. For those with clinically detectable lesions, the outreach camps provided same-day evaluation of the abnormalities, effectively eliminating multiple visits to different tiers of the healthcare system and the associated costs and time of doing so, all of which are associated with loss to follow-up [2, 7, 21–23]. The option of immediate surgical intervention following histologic confirmation was accepted by 24 of the 25 (96%) women to whom it was offered. In addition, those with breast cancer received an immediate referral for follow-up care.

To our knowledge this is the first breast care model of its type to be implemented in sub-Saharan Africa, inclusive of an option for next-day surgical intervention.

### Implementation challenges

There are several areas in which improvements can be made:

Logistics: (1) The overall turnout at each event exceeded our expectations (avg. >110 women/day; peak 171). We made adjustments by increasing the numbers of CBE nurses, radiographers, ultrasound machines and surgeons. Task-shifting administrative responsibilities (patient registration, data collection, etc.) to non-clinical personnel would allow healthcare providers the opportunity to focus solely on clinical issues. (2) Some women requested cervical cancer screening in addition to breast care services. Modifying the service platform to include cervical cancer screening would provide access to preventative, diagnostic and treatment services for the two most common cancers affecting women in Zambia and sub-Saharan Africa.

Packaging or bundling multiple services as we did requires the coordinated efforts and physical presence of many specialists in one place, and at the same time. The scarcity of mid- and high-level human resources in resource-constrained settings represents a potential limitation to bringing such a model to scale. However, human resource gaps can be filled using modern telecommunications technology, e.g., cytotechnologists at the point-of-care can be taught to prepare imprint cytology samples, identify and take digital images of the worst appearing areas on the glass slide, and electronically submit them to an offsite pathologist; ultrasonographers and nurses can be trained to perform ultrasound-guided biopsies, while being monitored using teleproctoring technology. Drones can be used to transport biopsy/cytology specimens to nearby pathology labs for final diagnosis.

Targeted Population: (1) The mean age of women attending the first outreach was 34.5 years (Table 1), whereas the majority of women in sub-Saharan Africa diagnosed with breast cancer are between ages 35–49 years. During the second outreach camp, we modified health promotion activities to target locations that would potentially increase access to older women, such as local churches. This resulted in an increase in the mean age of women in the second camp from 34.5 to 37.3 years (Table 1). (2) The breast cancer detection yield was relatively low. Of the women with ultrasound-confirmed breast masses (Kapiri– 11.8%; Kabwe– 12.5%), only 12.2% had histologically-confirmed invasive breast cancers. In studies of SSA women presenting to clinical care facilities with palpable breast masses, 16–34% were diagnosed with invasive breast cancer [24–27]. (3) Although most breast masses detected in women are not cancerous, they can seriously impact quality of life, and thus demand the serious attention of healthcare providers [28, 29]. Of the 48 women evaluated with core biopsy or needle aspiration, 87.5% had benign disease. There is a paucity of information regarding the prevalence of benign breast diseases in women who reside in resource-constrained settings [27, 30, 31]. In a recent retrospective study of a random sample of 365 women presenting to a walk-in breast specialist center in South Africa, over 50% were diagnosed with benign disease compared to 14% with breast cancer [32].

Targeted educational programs designed to increase breast cancer awareness and dispel myths and misconceptions, may increase the breast cancer detection yield of outreach efforts. Implementation of these programs in areas frequented by older women, such as local churches and markets, may increase the likelihood of their participation. Further facilitation of access through home-based clinical breast examinations with subsequent transport to clinical facilities (e.g. breast outreach camps) of those with detected abnormalities, may also increase the breast cancer yield. Lastly, partnering with local leaders and civic organizations to more deeply engage the community, in addition to mass media campaigns, could impact outcomes.

A clinical service platform that provides comprehensive breast care to the highest-risk individuals, differentiates between benign and malignant disease, and does so in a single-visit setting, may effectively reduce bottlenecks within an already strained healthcare system, and limit costly delays in care.

### Future directions

In order to determine the impact of a single-visit breast care algorithm and further define the magnitude of breast disease in Zambia, we strongly recommend the implementation of this approach on a larger scale, in both urban and rural settings. As with the cervical cancer prevention program scale-up [12], partnerships with the Ministry of Health and local and international stakeholders, in addition to leveraging and strengthening existing healthcare infrastructure while building upon the network of previously trained healthcare providers, will prove critical to its success. Continued use of the philosophy of adaptive innovation which, in principle, incorporates respect for local beliefs and culture while integrating new ideas and technology that fit the local circumstances, is mandatory.

Programmatic components should include (1) quality-assurance and quality control measures at all levels, (2) impact evaluation, (3) cost and cost-effectiveness evaluations (4) patient time-flow analysis (5) improved laboratory capacity, particularly the ability to perform immunohistochemistry and identification of significant tumor biomarkers and (6) implementation of strategies to decrease loss to follow-up among women receiving care, particularly those diagnosed with cancerous lesions.

## Conclusion

Over the next two decades breast cancer incidence and mortality will double across the globe. The impact on women residing in sub-Saharan Africa will be profound if effective and affordable approaches to early detection and treatment are not rapidly employed and brought to scale. The overall outcome of our single-visit model encourages its replication and scale-up to improve the quality of breast care in such settings.
